# Dual CNN for Relation Extraction with Knowledge-Based Attention and Word Embeddings

**DOI:** 10.1155/2019/6789520

**Published:** 2019-07-14

**Authors:** Jun Li, Guimin Huang, Jianheng Chen, Yabing Wang

**Affiliations:** ^1^School of Information and Communication, Guilin University of Electronic Technology, Guilin, Guangxi 541004, China; ^2^School of Computer Science and Information Security, Guilin University of Electronic Technology, Guilin, Guangxi 541004, China; ^3^Guangxi Key Laboratory of Trusted Software, Guangxi, China

## Abstract

Relation extraction is the underlying critical task of textual understanding. However, the existing methods currently have defects in instance selection and lack background knowledge for entity recognition. In this paper, we propose a knowledge-based attention model, which can make full use of supervised information from a knowledge base, to select an entity. We also design a method of dual convolutional neural networks (CNNs) considering the word embedding of each word is restricted by using a single training tool. The proposed model combines a CNN with an attention mechanism. The model inserts the word embedding and supervised information from the knowledge base into the CNN, performs convolution and pooling, and combines the knowledge base and CNN in the full connection layer. Based on these processes, the model not only obtains better entity representations but also improves the performance of relation extraction with the help of rich background knowledge. The experimental results demonstrate that the proposed model achieves competitive performance.

## 1. Introduction

Relation extraction (RE) [1–3] is the basis for the application of higher natural language processing, which has been widely used in important areas such as information retrieval [4, 5], knowledge graphs, representation learning, and textual understanding. RE can be simply regarded as a multiclass classification problem: given the sentence text of two entities, the relationship between the two entities is discriminated. For a pair of entities *e*_1_ and *e*_2_, the relationship between the two entities can be formalized by three tuples 〈*e*_1_, *r*, *e*_2_〉, in which *r* indicates the relation type. For instance, given a simple sentence containing an entity relationship, e.g., “*Bill Gates is the founder of Microsoft*,” the semantic relationship between the entities “*Bill Gates*” and “*Microsoft*” is “*founder.*”

Recently, deep learning has achieved good performance in natural language processing; thus, a large number of algorithms have adopted deep learning methods for feature extraction and RE. In 2012, Socher et al. [6] proposed using a recursive neural network (RNN) to solve the problem of relationship classification and obtained the representation of the sentence vectors through RNN for relationship classification. Zeng et al. [7] used a convolutional neural network (CNN) [8, 9] to combine word embedding and location information to extract relations. CNN can extract locally sensitive information from sentences represented by word vectors, obtain high-level features, and be effectively applied to relation classification and extraction. Currently, most CNN models for RE use the word vector in the sentence directly obtained from a single training model as the input and extract features. To remove the corpus richness limitation of the single-word vector training model, we use entity background knowledge as another CNN input, and then, we build a dual CNN structure by combining word embedding and entity background knowledge representation.

The attention mechanism [10, 11] was first applied in image processing, which can focus a neural network on the important target task information when processing image data. In natural language processing, the attention mechanism can effectively improve the effect of machine translation, specific target sentiment analysis, and other tasks. In RE, each word in a sentence has a different impact on a specific task; for example, in the sentence, “*the film is one of the year's best*,” the word “best” in the sentence plays a key role in indicating that the overall sentiment of the sentence is positive, and its importance is greater than that of the other words in the sentence. A neural network model based on an attention mechanism should identify which information in a sentence is important and focus on that information. Attention mechanisms have shown exceptional performance in sequence-to-sequence tasks and have achieved good results in sentence modeling. Lin et al. [12] proposed a sentence-level attention model to reduce the noise problem caused by false labels in the RE model. The attention weight matrix is used for high-level semantic representation, which improves the accuracy of sentence representation. However, these methods are still insufficient for characterizing the local and global information of entities in sentences. In our approach, through a knowledge-based attention mechanism, we obtain a representation of the relationships between the entity pairs in a current sentence and also the other relationships between the current entity pairs. These relationships help us to understand the relationship between the entity pairs in the current sentence.

The same pair of entities has different degrees of influence on different sentences in a knowledge base. For instance, the corresponding relationship between “*Bill Gates*” and “*Microsoft*” in the knowledge base is “*founder*.” The relationship label “*founder*” has a higher correlation to the sentence “*Bill Gates is the founder of Microsoft*,” than the sentence “*Bill Gates continues to serve on Microsoft's board as an advisor on key development projects*.” Therefore, in this paper, by introducing the attention mechanism of entity representation in a knowledge base, we can enrich the semantic background knowledge and improve the effect of RE.

## 2. Related Work

The main purpose of RE is to identify entities in text and extract semantic relationships between entities. Current mainstream RE techniques are divided into supervised learning methods and deep learning-based methods. A supervised RE system usually requires a large amount of manually labeled training data and automatically learns the corresponding extraction mode from the training data. Zheng et al. [13] proposed a method based on a kernel function. Poria et al. [14] proposed a method based on a 7-layer deep convolutional neural network to tag each word in opinionated sentences as either aspect or nonaspect word. Mintz et al. [15] proposed the distant supervision method and aligned New York Times news text with the large-scale knowledge graph, Freebase, which contains more than 7,300 relationships and over 900 million entities. Subsequently, many researchers proposed improvements to remote, distant supervision technology from various perspectives. Chen et al. [16] proposed a joint inference framework that employs such global clues to resolve disagreements among local predictions. Riedel et al. [17] enhanced the assumption of distant supervision. Takamatsu et al. [18] improved entity alignment technology, reduced data noise, and improved the overall effect of RE. The above distant supervision techniques assume that an entity pair corresponds to only one relationship. However, many entities have multiple relationships. Therefore, Hoffmann et al. [19] proposed using a multi-instance multilabel method to model RE and describe multiple relationships between entities pairs. Surdeanu et al. [20] also proposed a multi-instance multilabel method and Bayesian networks for RE. Taghva [21] described formal concept analysis (FCA) to identify and extract personal names and relationships, and FCA can decode text sequences by using the Viterbi algorithm used with hidden Markov models.

Recently, many researchers have begun to apply deep learning techniques to RE [22, 23]. Socher et al. [6] proposed using RNNs to solve RE problems; the method first parses a sentence and then learns the vector representation for each node on the syntax tree. Through the RNN, the method can start with the word vectors at the lowest end of the syntactic tree and iteratively merge the vectors according to the syntactic structure of the sentence. Finally, a vector representation of the sentence is obtained and used for relation classification [24–26]. This method effectively considers the syntactic structure information of the sentences, but at the same time, it cannot consider the position and semantic information of two entities in a sentence. Zeng et al. [27] used the word vector and the position vector of the word as input for the CNN and obtained the sentence representation through the convolutional layer, the pooling layer, and the nonlinear layer. By considering the location vector of the entity and other related lexical features, the entity information in the sentence can be used for RE. Bollegala et al. [28] also proposed a new CNN for RE that uses a new loss function, which can effectively improve the discriminability between different relationship categories. Luo et al. [29] proposed a deep learning model with a novel structure, and the attention mechanism is additionally utilized in an effort to assign weights of key issues in the network structure. Lin et al. [12] proposed a neural network model based on a sentence-level attention mechanism. The method can assign weights to each sentence of an entity pair according to a specific relationship. Through continuous learning, effective sentences are given higher weights, while noisy sentences are given lower weights. Currently, the RE of a neural network is mainly used for preset relation sets. However, open domain-oriented relational extraction is still a relatively traditional method based on templates. Therefore, in our method, we attempt to introduce a knowledge base into relational extraction as background knowledge to allow automatic discovery of new relationships and entities.

## 3. Methodology

### 3.1. Knowledge-Based Attention Model

**Figure 1:**
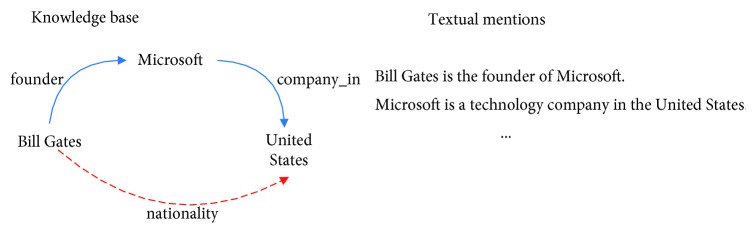
A knowledge base fragment and the expression of the entities in the texts.

Nickel et al. [30] introduced the terminology for the representation of knowledge bases, which are represented using RDF (resource description framework) triples in the form (*subject, relation,* and *object*); for instance, consider the knowledge base fragment and the expression of the entities in the texts shown in Figure 1, where the nodes indicate the entities and the relations are shown as directed labeled edges. For brevity, we denote triples by 〈*e*_*r*_, *r*, *e*_*o*_〉, in which *e*_*r*_ and *e*_*o*_ denote the subject and object entities, respectively.

**Figure 2:**
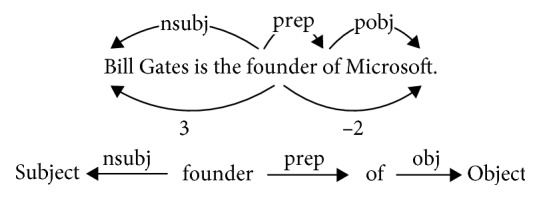
Textual relation representation and relative distances.

For the sentence “*Bill Gates is the founder of Microsoft*,” we can only obtain the pair of entities “*Bill Gates*” and “*Microsoft*” and the relationship “*founder*” between them, but we cannot obtain information about the relationship between “*Microsoft*” and the “*United States*.” However, in the knowledge base, the relationships between these entities are simply and clearly expressed. Therefore, our goal is to include the representation of the entity relationship in the knowledge base in the model input. To find entity mentions in the text, we first use the Stanford Named Entity Recognizer (NER) [31]. Each document can be segmented into sentences, and each token can be classified into four categories by the NER tagger. We treat consecutive tokens that share the same category as a single entity mention, and then, we associate the entities mentioned in the text with those in the knowledge base. To combine the textual information, we also use the Stanford Dependency Parser to represent the text, as illustrated in Figure 2, in which *nsubj* denotes the nominal subject, *prep* is the prepositional modifier, and *pobj* is the object of a preposition.

We use a CNN to extract the feature information of these entity relationships from the knowledge base. In the vector representation layer, we use the word embeddings and position embeddings as the input to the network. Word embeddings are distributed representations of words that map each word in the text to a low-dimensional vector that can be trained by Word2vec [32] or GloVe [33]. Position embeddings are important features in RE; they represent the distance between the entity pair and the relationship. Figure 2 shows the relative distances; the relative distances from the word “*founder*” to “*Bill Gates*” and “*Microsoft*” are “*3*” and “*–2*.”

**Figure 3:**
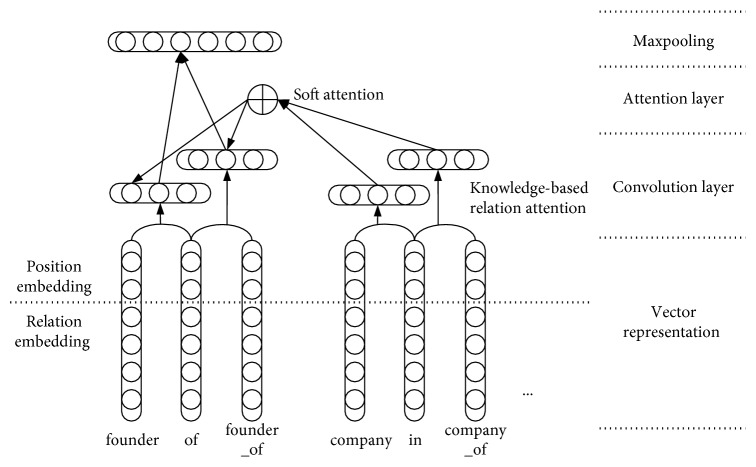
Knowledge-based attention model.

The knowledge-based attention aims to recognize and mine relation from the sentence or text; in our model, we embed both the word-level and relation representations. As in Figure 1, “founder_of,” as a single token, meanwhile, the word embedding of “founder” and “of” takes the relation as a sequence of words. In this paper, we define *r*={*r*_1_, *r*_|*n*|_}as a candidate relation chain, where |*n*| ≤ 2 is the number of relations in the candidate relation chain. Therefore, we combined the word embedding and the relation representation as the input. Similarly, the relation “company_of” between “Microsoft” and “United States” is represented as word embedding of “company” and “of” and the relation “company_of,” which comes from the knowledge base, and we hope to provide more information for current relation recognition through these relationships related to the current entities. The relation representation focuses more on the global information of the context. However, relation representation is often subject to the negative effects of data sparsity because some relationships may rarely appear in our data. After word embedding, converting “one-hot representation” into *d*-dimensional word vectors *V* ∈ *ℝ*^|*V*|×*d*^ and the relation embedding vectors *V*_relation_ ∈ *ℝ*^|*V*_relation_|×*d*^, where |*V*| and |*V*_relation_| are the vocabulary size and the number of relations in the knowledge base, respectively. Then, the output of the embedding layer is sent to the convolutional layer of CNN for feature extraction.  Figure 3 depicts the CNN architecture. Actually, there are many relationships related to current entities in the knowledge base, such as “father_of” and “place_of_birth.” Here, we only use the relationship “company_of” as an example. In the first layer, each word and its position information are mapped to a continuous representation using an embedding matrix *V* and the word embedded *e* is converted to the vector *v* by using the following formula:

(1) $$\begin{array}{c} v=Ve. \end{array}$$

In the hidden layer, we obtain the hidden layer features by a weight vector *W*, a bias vector *b*, and an activation function tanh, which are shown in the following formula:

(2) $$\begin{array}{c} h=\tanh\left(W^{-1}v_{-1}+W^{0}v_{0}+W^{1}v_{+1}+b\right), \end{array}$$

where *v*_0_ denotes the current word embedding vector and *v*_1_ and *v*_2_ denote the word embedding vector before and after the current word, respectively.

The knowledge-based attention model can mine the relationship representation of current entity pairs and also acquire relationship information for other entities related to the current entity in the knowledge base. As in Figure 1, in addition to acquiring the relationship between “*Bill Gates*” and “*Microsoft*,” we can also obtain the relationship between “*Bill Gates*” and the “*United State*s” and “*Microsoft*” and the “*United States*.” These relationships can add additional information about the entity pairs to the input text.

### 3.2. Dual CNN Model

In Section 3.1, we introduce the knowledge-based attention model, which can obtain additional information of the input entity pairs in the knowledge base. To obtain the word embedding information in the input text, we use another CNN to identify the sentence features. We adopt a piecewise CNN (PCNN), designed by Zeng et al. [27], to predict the relation. The network structure is similar to the knowledge-based attention model described above. To identify the importance of the words in the sentence, we calculate the correlation coefficient between each word in the sentence and its context vector and use the word vector and the context vector as the convolution input so that the words with a larger coefficient of relationship with other words in the sentence receive more attention.

Assume that the length of a sentence is *n*; *w*_*i*_ ∈ *R*^*k*^(1 ≤ *i* ≤ *n*) is the word vector representation of the *k* dimension corresponding to the *i*‐th word in the sentence. Let *m*_*i*_ be the context vector of *w*_*i*_; *m*_*i*_ is obtained by the weighted sum of multiple word vectors, which is shown in the following formula:

(3) $$\begin{array}{c} m_{i}=\overset{n}{\underset{j=1,j\neq i}{\sum }}\left(a_{i,j}w_{j}\right), \end{array}$$

where *a*_*i*,*j*_ is the weight obtained by the softmax function, as shown in the following formula:

(4) $$\begin{array}{c} a_{i,j}=\frac{\exp\left(\text{score}\left(w_{i},w_{j}\right)\right)}{\sum_{j\prime =1}^{n}\exp\left(\text{score}\left(w_{i},w_{j{}^{\prime }}\right)\right)}, \end{array}$$

where the *score* function is used to calculate the correlation coefficient between two words, which measures the correlation between words, as defined in the following formula:

(5) $$\begin{array}{c} \text{score}\left(w_{i},w_{j}\right)=v_{a}^{T}\;\tanh\left(W_{a}\left[w_{i}\;⊕\;w_{j}\right]\right), \end{array}$$

where *v*_*a*_ and *W*_*a*_ are the training parameters.

Considering that the correlation between two words in a sentence tends to weaken with an increase in distance, the distance attenuation factor *λ* can be introduced in formula (5), and the formula can be converted to the following formula:

(6) $$\begin{array}{c} \text{score}\left(w_{i},w_{j}\right)={\left(1-\lambda \right)}^{u}\cdot v_{a}^{T}\;\tanh\left(W_{a}\left[w_{i}\;⊕\;w_{j}\right]\right), \end{array}$$

where *λ* ∈ [0,1] and *u*=|*j* − *i*| − 1. When *λ* approaches 0, the correlation between the two words is almost unaffected by the distance factor, and when *λ* approaches 1, the correlation between the two words depends on the distance factor.

Through the word vector *w*_*i*_ and the context vector *m*_*i*_, the final word vector representation can be obtained and used for subsequent convolution operations, as shown in the following formula:

(7) $$\begin{array}{c} w_{i}^{\prime }=w_{i}\;⊕\;m_{i}. \end{array}$$

**Figure 4:**
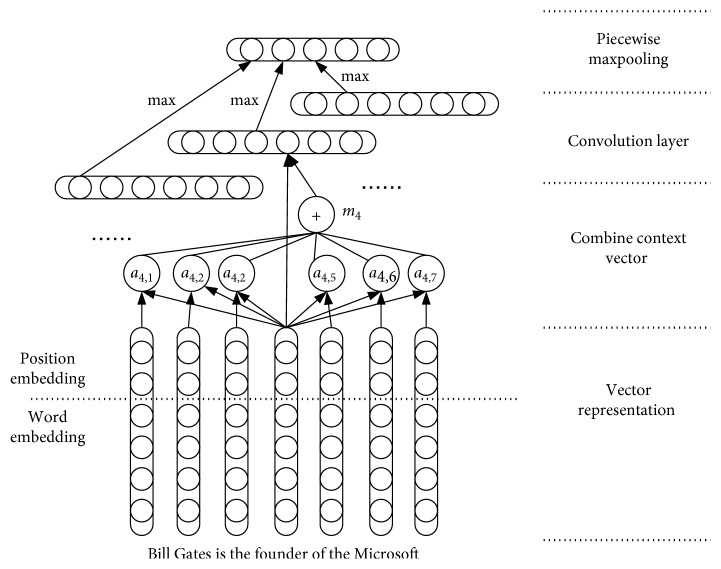
Text representation model based on CNN.

In Figure 4, we use the sentence “*Bill Gates is the founder of Microsoft*” as an example to illustrate the network structure. The weight between the word “*founder*” and the other words in the sentence is denoted as *a*_4,*j*_, and then, the context vector *m*_4_ of *w*_4_ is combined with its vector representation as the input to the convolution layer.

**Figure 5:**
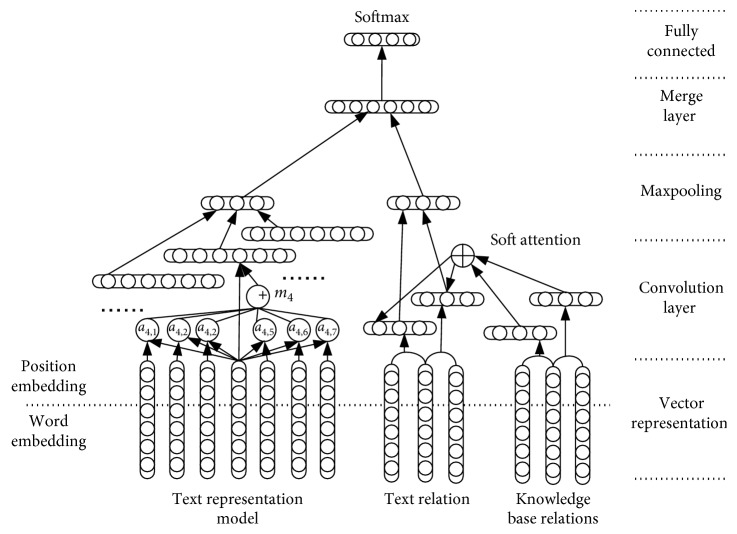
The architecture of the dual CNN.

We merge the above two networks to construct a dual CNN relational extraction model; each network has its own input layer, convolution layer, and pooling layer. Then, the layers are merged into the fully connected layer. The dual CNN architecture is shown in Figure 5.

In traditional relational extraction tasks, erroneous labels are inevitably introduced, which creates noise relational extraction. In this paper, we introduce the entity pair to the knowledge base as the attention mechanism. We reduce the noise by fully mining the correlation between the entity pairs in the knowledge base and the semantic information of the prediction sentences. For the set *S* of sentences containing the same entity pairs, the number of sentences is *n*; that is, *S*=(*s*_1_, *s*_2_,…, *s*_*n*_). To calculate the degree of correlation between the input sentence *s*_*i*_ and the relationship *r*, the attention matrix is obtained by calculating the inner product of the sentence vector and the correspondence vector of the entity pairs in the knowledge base. The weight matrix is calculated as shown in the following formula:

(8) $$\begin{array}{c} a_{i}=\text{soft}\max\left(s_{i}Ar\right), \end{array}$$

where *A* denotes the weighted diagonal matrix, *r* is the vector representation of the entity pair of the corresponding predictive relation *r* in the knowledge base, and *A* is obtained through random initialization in the train process. To assign greater weights to sentences that are more relevant to the relational vectors, the sentences of the corresponding entity pairs are represented as follows:

(9) $$\begin{array}{c} c=\underset{i}{\sum }a_{i}s_{i}. \end{array}$$

Finally, the relational label $\hat{y}$ of the sentence *s*_*i*_ is predicted from all relational sets *Y* by using the softmax classifier:

(10) $$\begin{array}{c} \hat{p}\left(y\;|\;s_{i}\right)=\text{soft}\max\left(Yc+b\right),\\ \hat{y}=\underset{y}{\arg\max}\hat{p}\left(y\;|\;s_{i}\right), \end{array}$$

where *b* is the bias vector, *s*_*i*_ denotes the current sentence vector, and $\hat{p}\left(y|s_{i}\right)$ denotes the probability of the entity pair belonging to relational label *y* in the current sentence *s*_*i*_.

### 3.3. Optimization Strategy

We use the cross-entropy cost function as the objective function, which is defined as follows:

(11) $$\begin{array}{c} J\left(\theta \right)=\overset{T}{\underset{i=1}{\sum }}\log\;p\left(r_{i}\;|\;H,\theta \right), \end{array}$$

where *θ* denotes all of the parameters in the model and *T* denotes the number of sentence sets, and then, the *Adam* optimizer is used for the parameter updates.

To prevent model overfitting, dropout is used for the regularization constraints in each forward propagation, and some hidden layer node features are randomly discarded; i.e., weight updating does not depend on the interaction of the fixed nodes. In addition, this paper adopts L2 regularization, which is multiplied by a factor *λ* less than 1 during iteration to reduce the value of the parameter *θ*. The regularization operation reduces the influence of data offset on the result, enhances the antidisturbance of the model, and avoids overfitting.

## 4. Experiments

### 4.1. Data Availability

The experiment data used to support the findings of this study have been deposited in the GITHUB repository https://github.com/mrlijun2017/Dual-CNN-RE.

To evaluate the dual CNN attentional RE model, we used the dataset developed by Riedel et al. [17] in 2010. The dataset is generated by matching the knowledge base Freebase and the New York Times (https://catalog.ldc.upenn.edu/LDC2008T19) text set [34] through heuristic aligning, which is widely used in RE. Specifically, this paper uses sentences from 2005-2006 in the corpus as the training data, and the testing data are aligned to the year 2007. The dataset contains 53 relations (“*NA*” denotes no relation between entity pairs), the number of entities in the training set is 281, 270, and the number of entities in the testing set is 96 678.

The average precision (*P@N*) and the precision-recall (*P-R*) curve are used to evaluate the effectiveness of our method. The algorithm is evaluated by comparing the accuracy of the top *N* terms and the area covered by the *P-R* curves.

To verify the expressiveness of our model in sentence relation classification, we use three open datasets (http://cogcomp.cs.illinois.edu/Data/QA/QC/), SST-1, SST-2, and TREC to conduct the experiments. The relevant information for these three datasets is shown in Table 1.

### 4.2. Experimental Results and Analysis

#### 4.2.1. Influence of Distance Attenuation on the Model

**Figure 6:**
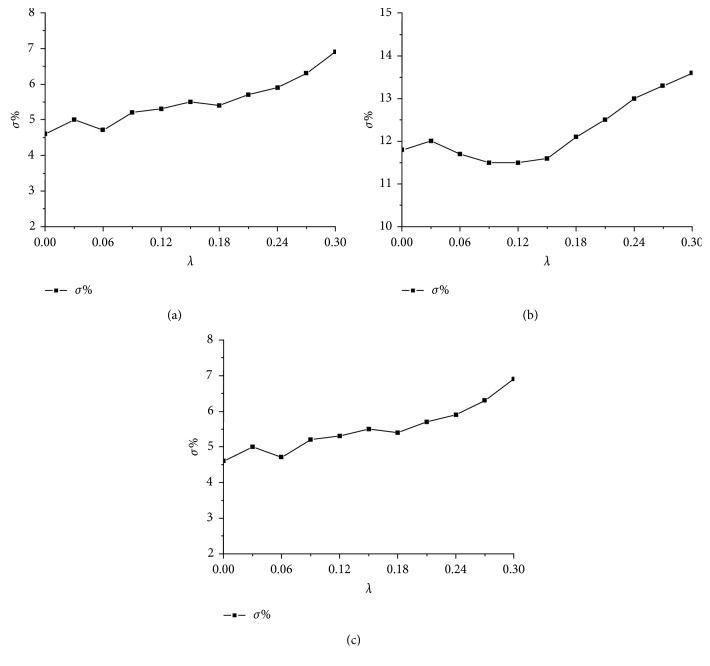
Effect of distance decay on the generalization ability of the model. (a) Dataset SST-1. (b) Dataset SST-2. (c) Dataset TREC.

The introduction of distance attenuation is an extension of the calculation of the correlation coefficient between words. It can express the influence of the distance factor between words on the correlation to more accurately describe the correlation between two words. The selection of the distance attenuation factor determines the distance factor between words. The magnitude of the correlation influences the effect of the sentence relation classification to a certain extent. To obtain the appropriate values of the model for each dataset, the degree of influence of the exponential distance attenuation on the sentence correlation calculation in equation (6) is limited; *λ* ∈ [0,0.3] is selected using the error rate *σ* as the evaluation index. The experimental results are shown in Figure 6.

In Figure 6, we can see that the effect of *λ* on the generalization ability of the models is not consistent for the datasets with different tasks. For datasets SST-1, SST-2, and TREC, when *λ* is 0.09, 0.09, or 0.12, respectively, the generalization ability of the model is the best. For the datasets SST-1 and SST-2 with a longer average sentence length, introducing appropriate distance attenuation can allow more accurate correlation coefficients between words to be obtained through model training, thus improving the classification performance. For the TREC dataset with a shorter average length, there is a strong correlation between the words in a sentence, and a good classification effect can be achieved when the distance attenuation factor is 0 or a small value. However, as the distance attenuation introduced is exponential, with an increase in *λ*, the influence of the distance factor on the correlation between words will rapidly increase. The corresponding word vectors near each word tend to obtain more attention weight in the context vector, which causes the generalization ability of the model to gradually decline.

#### 4.2.2. Influence of Attention on the Model

In this section, we first introduce some parameter settings in the experiment, and the parameter settings refer to the experience of Ji et al. [35]. We select the dimension of the word embedding *d*_w_ among [1 and 300], and the dimension of the position embedding *d*_p_ among {5, 10, and 20}. In our experiment, we set *d*_w_=50 and *d*_p_=5, the batch size is 50, the learning rate is *η*=0.001, and the regularization superparameter is *λ*=0.0001.

To verify the improvement of the knowledge-based attention model for RE, we compare the results of the word embedding model and the knowledge-based attention mechanism model. Table 1 shows the accuracies of the two models for the top 100, top 200, and top 300 extracted relation instances. In Table 2, we can see that, compared with the single-word embedding model, the KB attention mechanism model improves the accuracy of the RE.

In addition, five other published methods were selected for comparison. *Mintz* was proposed by Mintz et al. [15] and uses all instances to extract features. Hoffmann et al. [19] adopted the method of multi-instance learning, called *MultiR*. Surdeanu et al. [20] proposed the method of multi-instance multilabels called *MIML*. PCNN_ATT was proposed by Lin et al. [12], which adds the sentence attention mechanism to the model. Gated recurrent unit with attention (GRU_ATT) is the new method proposed by Cai et al. [36]. In terms of the performance, our method produces better results than the GRU_ATT method. Compared with the sentences vector obtained using GRU, we believe that CNN is better than GRU in extracting local features.

**Figure 7:**
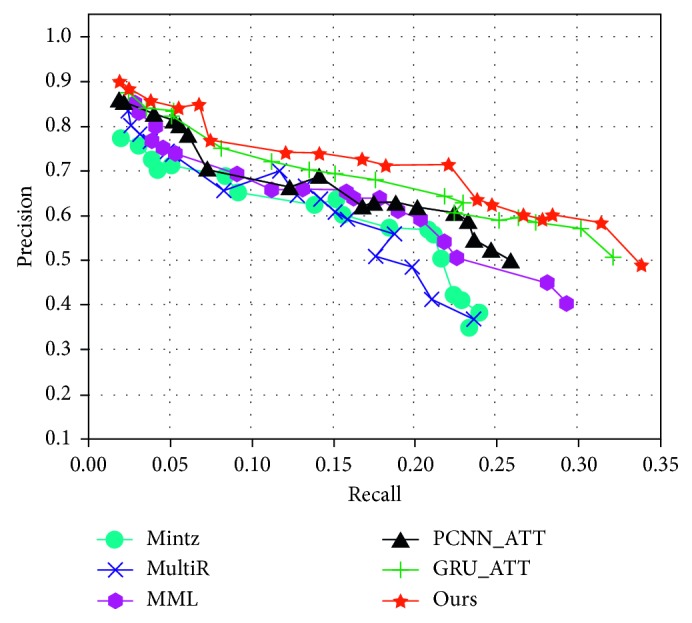
Aggregate precision/recall curves for a variety of methods.

We re-implement this part of the experiment through the methods and datasets in the relevant papers and compare them with our methods.  Figure 7 shows the aggregate precision/recall curves for our method and other prior approaches. In Figure 6, we can see that our approach outperforms the other approaches, and the recall can achieve 0.34, which is higher than that (0.32) of GRU_ATT. Overall, the precision curve of our approach is better than that of the other approaches.

**Figure 8:**
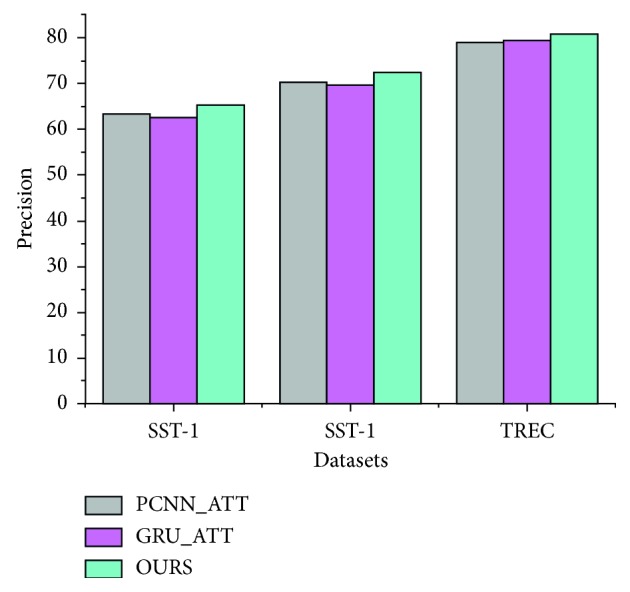
Comparison of the model performances for the datasets.

We also compare the performance of the models PCNN_ATT and GRU_ATT and our model on the = SST-1, SST-2 and TREC datasets, which is a task of sentence relation classification, and the purpose of that is whether the direct use of word vectors and relation attention in the knowledge base can affect the classification of sentence relation. And the input of this task is word vectors and the relation representation of sentences, while the output is the relation label. The experimental results are shown in Figure 8.

Compared with the other two models, in our model, each word embedding vector is separately convoluted and pooled, and feature fusion is performed at a higher level, which avoids the feature limitation of the single-word vector training model and results in more abundant extracted features. Meanwhile, the word vector attention mechanism is introduced into our model, which makes it easier to extract key information from sentences. Our model combines the advantages of attention mechanisms and dual CNN to further improve the accuracy of sentence relation classification.

## 5. Conclusions

We use word embedding and entity embedding of a knowledge base as the CNN input and propose a dual CNN RE model based on a knowledge-based attention mechanism. Entity embedding can provide more background knowledge to predict relations, and word embedding can obtain more sentence features due to the attention mechanism. Experiments show that our proposed model outperforms previous methods and is suitable for entity RE tasks. We also use our model for sentence classification tasks, and our model also has a better performance. In the future, we will attempt to use multiclass models to represent sentence vectors, improve attention mechanisms, and apply the models to other text understanding tasks. How to quickly learn new relationships and examples from existing neural network models is also a practical problem worth exploring.

## Figures and Tables

**Table 1:** Relevant dataset information.

Datasets	Average sentence length	Number of sentences	Number of sentences in the test set
SST-1	18	11855	2210
SST-2	19	9613	1821
TREC	10	5952	500

**Table 2:** Precision values for the top 100, 200, and 300 extracted relations.

Accuracy (%)	Top 100	Top 200	Top 300	Average
Word embedding	0.74	0.72	0.64	0.70
Knowledge-based attention model	0.83	0.79	0.72	0.78

## Data Availability

The experiment data used to support the findings of this study have been deposited in the GITHUB repository https://github.com/mrlijun2017/Dual-CNN-RE.
